# Injections of Nitrate of Silver in Dysentery

**Published:** 1850-07

**Authors:** 


*Injections of Nitrate of Silver in Dysentery.* By J. W. Richardson, M. D., of Rutherford Co., Tenn.

Dr. Richardson said that he wished to direct the attention of the Society to the use of injections of nitrate of silver in malignant cases of dysentery. He had first used it in Sept. 1848. The patient was a delicate female, mother of several children, who had been laboring under a severe form of dysentery for five or six days, and who had been well treated in the usual manner by an intelligent physician. All the usual remedies having failed to afford any relief, Dr. R. said that on his second visit to the patient, whilst thinking of the case, and having witnessed the unsuccessful use of many remedies, the inflammation and ulceration of the mucous surface of the large bowels characteristic of dysentery, suggested the nitrate of silver as the very remedy to relieve them. Upon his arrival, he found the patient worse than she was the day before. The discharges were very frequent, consisting of large quantities of dark, gangrenous looking mucus, and more offensive than any he had ever smelled. The woman was nearly pulseless—every discharge produced the most deadly sickness, and she could not be turned on the bed without approaching syncope. Dr. R. advised the nitrate of silver, an injection of which was prepared, about 15 grs. to 4 ounces of water, by the attending physician, and thrown up the rectum. This remained some six hours before it was thrown off, and the bowels were quieted until the next morning, when, as there seemed to be a tendency to a return of the dysenteric symptoms, the remedy was repeated. The disease was arrested immediately, and the patient recovered. Dr R. said the result of this case, and the speedy and effectual relief afforded by the nitrate of silver, filled him with astonishment and pleasure:—*astonishment*, because he had never before thought of the remedy in intractable cases of dysentery, when he had so often witnessed its remedial virtues in inflammations and ulcerations of various mucous surfaces; and *pleasure*, because he thought that he had made a grand and important discovery! But he said, when reading Braithwaite's Retrospect a few evenings afterwards, he found that the remedy had been used in Europe in similar cases, before he had ever thought of it. See Braithwaite's Retrospect, part 16, p. 156, c. 7, where you will find some valuable facts recorded in favor of the use of nitrate of silver, not only in dysentery, but also in the troublesome, frequent and painful diarrhea in typhoid fevers, and in the protracted diarrhea of young children. He thought the remedy might be used in much larger doses.

Dr. R. had used the remedy since, in several cases, after the usual course of treatment had failed, where the patients were completely prostrated, and invariably with success. He used it in about the proportion of 10 grs. to 4 and 6 ounces of rain water, if the latter could be obtained, if not, he used common spring or well water; and he had also combined laudanum, and sulph. morph. with the nitrate, but could not say that the smarting or burning was prevented by the combination. The pain produced by the injection was not *always* sufficient to make the patient complain, though it did sometimes. He thought that he had used as much as 15 or 20 grs., or that this quantity had been used by his advice in one case. He did not desire to fix the precise quantity of the nitrate, nor water to be used in the enema, so much as to direct the Society to the remedy, and to solicit their experience.

Dr. Avent said that having heard of the use of nitrate of silver as an injection in dysentery, he had tried it in the worst case of dysentery he had ever seen, and which had resisted all treatment for five days. He said he gave only one enema, composed of 20 grs of the medicine to 4 ounces water, which afforded immediate relief, the griping and purging ceasing instantly. He had also used it in another case, not so bad a case, however, but with marked relief.

Dr. Gordon said that he was not surprised at all at what he had heard from Drs. R. and A. as to the efficiency of nitrate of silver, in malignant cases of dysentery. He was prepared to believe any thing almost as to the remedial effects of this article in diseases of mucous surfaces—indeed it was the greatest of all remedies. But he had been in the habit of treating dysentery in a different way altogether to the one commonly pursued. He converted the dysentery into a diarrhea by giving Epsom salts in small and separate doses, and the patient scarcely ever needed any thing else. He scarcely ever gave any other medicine. The diarrhea ceased of itself by withholding the salts, and the patient got well.—*Proceedings of the Tennessee Med. Society.*

